# Hemodynamic Perturbations in Deep Brain Stimulation Surgery: First Detailed Description

**DOI:** 10.3389/fnins.2017.00477

**Published:** 2017-08-28

**Authors:** Tumul Chowdhury, Marshall Wilkinson, Ronald B. Cappellani

**Affiliations:** University of Manitoba Winnipeg, MB, Canada

**Keywords:** deep brain stimulation, Parkinson disease, cardio-vascular changes, sub-thalamic nucleus

## Abstract

**Background:** Hemodynamic perturbations can be anticipated in deep brain stimulation (DBS) surgery and may be attributed to multiple factors. Acute changes in hemodynamics may produce rare but severe complications such as intracranial bleeding, transient ischemic stroke and myocardium infarction. Therefore, this retrospective study attempts to determine the incidence of hemodynamic perturbances (rate) and related risk factors in patients undergoing DBS surgery.

**Materials and Methods:** After institutional approval, all patients undergoing DBS surgery for the past 10 years were recruited for this study. Demographic characteristics, procedural characteristics and intraoperative hemodynamic changes were noted. Event rate was calculated and the effect of all the variables on hemodynamic perturbations was analyzed by regression model.

**Results:** Total hemodynamic adverse events during DBS surgery was 10.8 (0–42) and treated in 57% of cases.

**Conclusion:** Among all the perioperative variables, the baseline blood pressure including systolic, diastolic, and mean arterial pressure was found to have highly significant effect on these intraoperative hemodynamic perturbations.

## Introduction

Deep brain stimulation (DBS) has become an established surgical therapy for patients with Parkinson's disease (PD) who are refractory to standard medical management, as well as being used for other chronic neurological conditions (Shindo et al., 2013). This procedure requires precise stimulation of different thalamic and sub thalamic nuclei, intraoperative neurological monitoring and patient's cooperation to perform certain neurological examinations (Camerlingo et al., 1990).

Hemodynamic perturbations can be anticipated in this type of surgery due to a number of reasons including age related factors, anxiety, semi sitting position, surgical procedure (electrode insertion), presenting disease (autonomic dysfunctions), presence of other co morbidities and effect of concurrent medications (Haapaniemi et al., 2001; Mata et al., 2012; Vigneri et al., 2012). The effects of stimulation of different thalamic and sub thalamic nuclei on cardiovascular changes are still a matter of investigation (Jain et al., 2012; Zrinzo et al., 2012). A recent study highlighted that cardiovascular changes (carotid stenosis or ECG changes) usually precedes motor symptoms or these may be independent predictors of PD (Chakrabarti et al., 2014). Added to these, the use of anesthetic agents for conscious sedation can also affect the hemodynamic changes adversely (Green et al., 2010). Acute changes in hemodynamics may produce some unwanted complications such as intracranial bleeding, transient ischemic stroke, myocardium infarction etc. (Hyam et al., 2012). However, the literature revealing hemodynamic disturbances in this type of surgery is still largely unknown.

Therefore, this retrospective study attempts to determine the incidence of total hemodynamic perturbances (rate) and related risk factors in patients undergoing DBS surgery.

## Materials and methods

After the local institutional ethics committee [HS15730] approval, all patients undergoing DBS surgery from April 1, 2000 to July 31, 2012 were recruited for this study. For retrieving the data, the Canadian Classification of Health Interventions (CCI) codes (1.AN.53.SZ.JA or 1.AN.53.SE.JA) and the International Classification of Diseases (ICD) code (ICD-9-CM code 02.93) were used. Demographic characteristics including patient's characteristics, disease and risks factors characteristics, procedural characteristics and intraoperative hemodynamic changes were noted. Event rate (total hemodynamic perturbations in relation to total anesthesia time) was calculated and the effect of all the variables on hemodynamic perturbations was analyzed by regression model.

Hemodynamic perturbations- These are defined (number of episodes) as below. Hypotension- less than 90 mm Hg systolic BP:

Hypertension-more than 140 systolic and 90 mm Hg of diastolic BP
Bradycardia-Heart rate less than 50 bpm
Tachycardia-Heart rate more than 90 bpm
Any ECG changes other than normal sinus rhythm

These events were noted during two-phases: first during electrode (nuclei) stimulation, and second-during the battery placement. During the electrode stimulation, hemodynamic events were again noted in two phases, specifically, the right and left sided electrode (nuclei) stimulation related. The total hemodynamic events were calculated as events during electrode stimulation plus events during battery placement. To match the accuracy of data, neurophysiologist was asked to provide the time at which he started to stimulate the electrode (nuclei) and hemodynamic events during 20 min from the start of simulation time (as reported by the neurophysiologist) was taken for the calculation purpose. Events were noted every 5 min.

### Anesthesia protocol

Standard monitors including EKG, pulse oximetry and invasive blood pressure monitoring (IBP) were applied and for the initial phase (placement of burr hole) of DBS surgery, all patients received monitored anesthesia care (midazolam 1–2 mg or/and propofol 25–50 mcg/kg/min and/or remifentanil 0.02–0.05 mcg/kg/min and/or fentanyl 25–50 mcg bolus). The infusions were stopped approximately 30 min before the actual testing and re-started once the testing was completed.

For the battery placement procedure, all patients were given general anesthesia with tracheal intubation. As per the anesthesiologist discretion, the standard induction technique involved combination of remifentanil/fentanyl/sufentanil plus propofol plus rocuronium. The anesthesia was maintained with volatile anesthetics (desflurane or sevoflurane) and opioids as needed. At the end of procedure, the muscle relaxant was reversed with the reversal (neostigmine and glycopyrrolate) and the trachea was extubated after ascertaining four twitches on train of four monitor as well as full recovery of consciousness. All the patients were transferred to post-anesthesia care unit for further observation.

### Surgery protocol

The DBS procedure was performed either in one stage (both the electrode placement and the battery placement on the same day) or two stage (the battery placement on other day). The same surgeon performed all the procedures from 2003 till 2012. On the morning of the operation, a rigid head frame (Leksell) was placed on the patient by the surgeon utilizing local anesthesia (0.25% bupivacaine 5–10 ml) and magnetic resonance imaging was performed in order to delineate the x, y, and z coordinates of defined structures. After this, the patient was transferred to the operating room where he/she was positioned in semi-sitting position. The stimulation involved certain thalamic/subthalamic nuclei [ventralis intermedius nucleus (VIM), the sub-thalamic nucleus (STN), and the globus pallidus (GPI)]. The whole procedure consisted of three parts: first, localization; second, insertion of electrodes and stimulation, and third, internalization of leads and battery placement. The first two procedures were done under monitored anesthesia care and third procedure (battery placement) was performed under general anesthesia.

### Neurophysiology protocol

After scalp opening and burr hole placement, microelectrode recordings were carried out using the FHC electronic Microdrive and micro/macro electrode (Frederic Hare Corp., USA) and the Lead point recording/stimulation system (Medtronic Corp.). Target acquisition was obtained from AC-PC coordinates derived from preoperative MRI sequences in conjunction with the Leksell frame system and a custom targeting software. Microelectrode impedances were acceptable between 0.5 and 2 MΩ. A survey of electrical activity was conducted from 10 mm above to 5 mm below the putative target. The depths of the top of the target nucleus (usually STN) and the bottom were noted and the appropriate changes in aggregate cellular activity observed for transitions into and out of the target nucleus. Monopolar test stimulation was conducted for each electrode trajectory above and below the putative target. The stimulus parameters were pulse duration of 0.06 ms, frequency of 130 Hz and stimulus amplitude ranging from 0.1 to 5 mA. During test stimulation, relief of parkinsonian symptoms was noted as well as any side effects due to activation of neighboring structures, the most common of which was the internal capsule or sensory apparatus. Stimulus thresholds for evoking side effects were noted and compared to the clinical benefit. Generally, a side effect produced by stimuli of ≤ 3 mA indicated unacceptable electrode proximity and an alternate trajectory was conducted.

### Statistical analysis

**Figure 1:**
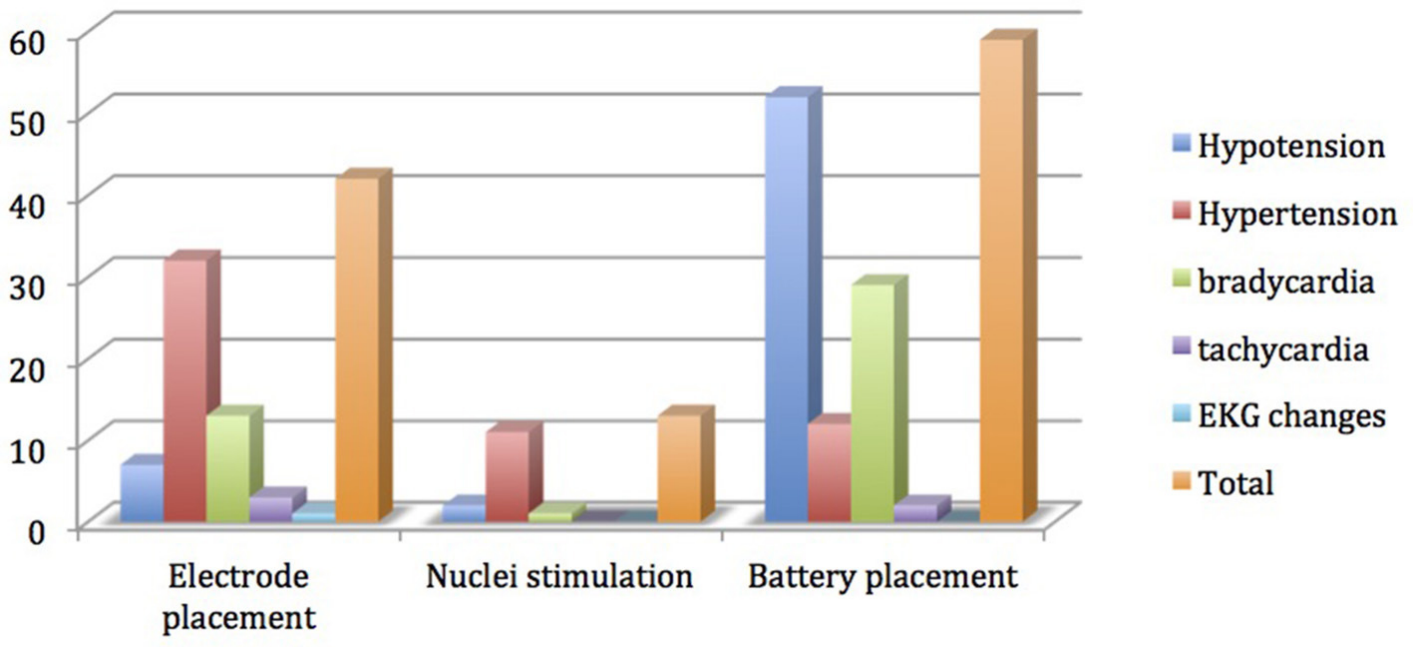
Distribution of hemodynamic events among various steps of deep brain stimulation surgery.

Rate ratios and their confidence intervals were estimated via negative binomial regression models. SAS version 9.3 (SAS Institute, Cary NC) was used for all analyses. Since each subject was under anesthesia for varying lengths of time, we did not compare the raw number of events during surgery between groups. Instead, we calculated event rates with minutes of anesthesia as the denominator, and used this as the outcome. The raw numbers were used to describe various hemodynamic events only in Figure 1. The continuous variables are reported as mean, min-max, and standard deviation. The *p*-value less than 0.05 is considered as statistically significant for this study purpose.

## Results

Data from 79 procedures were included for the final analysis. Among various characteristics noted, male patients (64.6%), Parkinson disease (50.6%), history of smoking (25.3%), hypertension (33%), bilateral electrode placement (73.4%) and same day battery placement (58.2%) were found to be more common variables in their respective groups (Table 1). Total hemodynamic adverse events during DBS surgery was 10.8 (0–42) and treated in 57% of cases. Baseline blood pressure including systolic, diastolic and mean arterial pressure was found to have highly significant effect [14, 31, and 19% greater chance of adverse hemodynamic event per 10 mm Hg increase in value respectively] on intraoperative hemodynamic perturbations (Table 2). DBP had the greatest impact among all the hemodynamic parameters. Other variables including type of disease, duration of symptoms, number of medications used, type of nuclei stimulated, laterality of DBS implants and battery placement on the same day had no significant effect on hemodynamic perturbations during DBS surgery (Table 3). The distribution of hemodynamic events among various steps of DBS surgery is shown in Figure 1. It is evident that most of the hemodynamic events were noted during battery placement. The hypotension was the most common hemodynamic perturbation observed during battery placement while hypertensive episodes were common events during both electrode placement and nuclei stimulation. Two patients had intracranial hemorrhage and one patient developed ST elevation during the electrode placement. No mortality was noted during the procedure.

**Table 1:** Demographic characteristics of patients.

**Characteristics**	**Frequency/Mean (*n* = 79)**	**Percentage/Range**
Age (years)	57.0	21–80
**GENDER**		
Male: Female	51: 28	64.6: 35.4
BMI	28.0	19.5–57.5
**ASA GRADE**		
1	10	12.6
2	36	45.6
3	33	41.8
**COMORBIDITIES**		
Hypertension	33	41.8
Diabetes	14	17.7
Coronary disease	7	8.9
Asthma	3	3.8
**RISK FACTORS**		
Smoking	20	25.3
Alcohol	13	16.5
OSA	7	8.9
**MAJOR DIAGNOSES**		
Parkinson	40	50.6
Essential Tremor	25	31.7
Dystonia	10	12.6
Multiple sclerosis	4	5.1
Duration of symptoms (years)	13.0	3–47
Number of Medications	3.4	0–8

*OSA, Obstructive sleep apnea*.

**Table 2:** Disease and procedure related characteristics.

**Characteristics**	**Frequency (*n* = 79)**	**Percentage**
**TYPE OF NUCLEI STIMULATED**		
STN	40	50.6
VIM	30	38.0
GPi	9	11.4
**DBS IMPLANTS SITE**		
Unilateral	21	26.6
Bilateral	58	73.4
**BATTERY PLACEMENT DAY**		
Same	46	58.2
Other	33	41.8
Complications	32	40.5
**BASELINE HEMODYNAMICS (mm Hg)**		
Systolic BP	140.0	104–200
Diastolic BP	75.4	54–110
Mean BP	95	65–140
Total no of events	10.8	0–42
Drugs for hemodynamic	45	57.0
Total duration surgery (min)	384.8	200–590
Total duration anesthesia (min)	451.1	260–630
Total duration battery (min)	116.3	70–194

*STN, Sub thalamic nucleus; VIM, Ventral intermediate, GPi, Internal globus pallidus; BP, Blood pressure*.

**Table 3:** Regression model; event rate predicated by various demographic variables.

**Factor**	**Rate ratio**	**95% confidence interval**	***p*-value**
Age per 10 y	1.16	0.96-1.40	0.13
**Sex**			
F/M	0.90	0.54–1.51	0.69
BMI per 5 Kg	0.91	0.74–1.11	0.38
Duration of symptoms (Per 10 y)	0.95	0.73–1.23	0.70
**ASA GRADE**			
Gr1/Gr2	1.28	0.60–2.70	0.52
Gr1/Gr3	0.78	0.36–1.65	0.51
Gr2/Gr3	0.61	0.36–1.01	0.05
**COMORBIDITIES (ABSENCE/PRESENCE)**			
Hypertension	0.63	0.39–1.02	0.06
Diabetes	1.05	0.56–1.98	0.88
**RISK FACTORS**			
Smoking	1.32	0.76–2.31	0.34
Alcohol	0.95	0.50–1.83	0.89
**MAJOR DIAGNOSES (ABSENCE/PRESENCE)**			
Parkinson	0.71	0.44–1.16	0.18
Essential tremor	1.06	0.63–1.79	0.82
Dystonia	1.66	0.74–3.72	0.24
Multiple sclerosis	1.59	0.51–4.92	0.44

## Discussion

Cardiovascular changes in DBS surgery are complex and multifactorial in origin. Three common factors can be delineated as the plausible causes. First, the neurological diseases including PD, multiple sclerosis (MS), and essential tremors may present with autonomic dysfunctions and these changes can be detected by various parameters including variability in heart rate (R-R variability), postural changes in blood pressure, Valsalva maneuver, cold pressor test, head-up tilt test, and other continuous robust monitoring methods; however, being a retrospective study, we just presented the hemodynamic parameters (Acevedo et al., 2000; Micieli et al., 2003; Jain and Goldstein, 2012). Secondly, the semi-sitting position combined with anesthetics may produce negative hemodynamic changes (Cicolini et al., 2011). And thirdly, the use of sedation as well as general anesthesia itself can also cause these perturbations. In addition, there are other contributing factors including procedure, side effects of anti-Parkinson medications, anxiety, pain, fatigue, and pre-existing comorbidities (diabetes, hypertension etc.) (Nicholson et al., 2002). We tried to note the effect of various pre/intraoperative variables on hemodynamic perturbations; however, our study did not show any association with these.

The main finding of our present study is that out of 79 DBS procedures, approximately 82% showed hemodynamic events, and there were approximately 11 hemodynamic events per DBS procedure. Importantly, more than half of these events were treated, therefore suggesting clinically significant cardiovascular alterations. On the regression analysis model, only the pre-operative blood pressure and its all components (SBP, DBP, and MAP) have shown significant association with these hemodynamic perturbations (Table 4). Among all the components, the DBP shows the greatest association for causing adverse hemodynamic events. A study by Tsukamoto et al. has shown that patients with PD can present wide fluctuations in blood pressure readings (more than 100 mm HG difference) in a day, and contrary to common observation of orthostatic hypotension in such disease, these patients may also experience very high SBP (200 mmHg or more) (Tsukamoto et al., 2013). Therefore, it is imperative to stabilize the blood pressure swings in such patients.

**Table 4:** Regression model: event rate predicted by laterality of procedure and day of the procedure.

**Factor**	**Rate ratio**	**95% confidence interval**	***p*-value**
**TYPE OF NUCLEI**			
GPi/STN	0.57	0.25–1.31	0.19
GPi/VIM	0.70	0.30–1.63	0.40
STN/VIM	1.21	0.72–2.03	0.46
**BASELINE HEMODYNAMICS (PER 10 mmHg)**			
Systolic BP	1.14	1.03–1.28	**0.01**
Diastolic BP	1.31	1.08–1.60	**0.01**
Mean BP	1.19	1.02–1.37	**0.02**
Baseline HR	1.23	0.94–1.60	0.11
**LATERALITY OF DBS IMPLANTS**			
Unilateral/Bilateral	0.99	0.57–1.72	0.98
**BATTERY PLACEMENT DAY**			
Same/Other	0.84	0.51–1.37	0.49

*STN, Sub thalamic nucleus; VIM, Ventral intermediate; GPi, Internal globus pallidus; BP, Blood pressure*.

One of the most serious complications of DBS surgery is intracranial bleed and its incidence can vary from 0.5 to 5% (Jain et al., 2012). Though it is a rare event it can be associated with permanent neurological deficit or even death (Jain et al., 2012; Wang et al., 2017). In our study, two patient developed neurological deficits due to intracranial hemorrhage. In a large case series and systemic review, age and hypertension are linked with increased incidence of intracranial bleed during functional neurosurgery (Sansur et al., 2007; Jain et al., 2012). In our study, approximately 75% of the patients having battery placement, 53% during electrode placement and 13% during nuclei stimulation showed hemodynamic perturbations. Strikingly, procedures involving electrode placement and nuclei stimulation were commonly associated with hypertensive episodes. One patient who had an intracranial bleed in our study showed hypertensive episodes during both the electrode placement and the stimulation phases. The mechanism of hypertension is not clearly understood yet; however, in a small case series (Green et al., 2010), the precise stimulation of periaqueductal gray matter incited cardiovascular changes including BP and HR changes (Hyam et al., 2012). Similarly, the STN stimulation may also cause hemodynamic perturbations that include a rise in HR (25 bpm) and BP (20 mm Hg). Our study also supports these findings. However, the laterality of these stimulations does not effect these changes (Sauleau et al., 2005) as also noted in our study. On the other hand, the battery placement was linked with more hypotensive and bradycardia episodes. These negative hemodynamic changes could be due to the combined effect of general anesthesia and autonomic dysfunctions related to neurodegenerative diseases. In our study, the day of battery placement did not reveal any association with such adverse events.

### Limitation

This is a retrospective study and the clinical correlation of the findings would be more justifiable if a prospective study could be done with a continuous hemodynamic monitoring during various surgical steps. Further to this, tests to detect autonomic changes can be applied to detect the actual nature and cause of these hemodynamic events.

## Conclusion

This study is the first detailed description of hemodynamic perturbations associated with DBS surgery in relation to influencing preoperative and intraoperative factors. Among all the factors, the baseline blood pressure does significantly affect the hemodynamic perturbations during the procedure and the DBP component has the highest impact on these events. Management of preoperative as well as intraoperative blood pressure is crucial to prevent major catastrophes during DBS procedure.

## Disclosure

This study abstract was submitted to 41st Annual Meeting of the Society for Neuroscience in Anesthesiology and Critical Care, San Francisco, CA and subsequently published in Journal of Neurosurgical Anesthesiology. 25(4): 440-501, October 2013. This abstract was also presented at Canadian anesthesia society meeting 2015, Ottawa.

## Ethics statement

This is a retrospective study and Ethics approval was given by Ethics committee, the University of Manitoba and Health Sciences Center [HS15730], Winnipeg, Canada.

## Author contributions

TC is the primary author who has assisted substantially in developing the hypothesis, collecting and interpretation of the data, writing and editing the manuscript. MW has assisted in writing and providing the neurophysiological monitoring data. RC has assisted in developing the concept, writing and editing the manuscript.

### Conflict of interest statement

The authors declare that the research was conducted in the absence of any commercial or financial relationships that could be construed as a potential conflict of interest.
